# Artificial intelligence and its application in early oral cancer screening: a systematic review

**DOI:** 10.3389/fonc.2026.1789708

**Published:** 2026-03-20

**Authors:** Weibo Huang

**Affiliations:** School & Hospital of Stomatology, Wuhan University, Wuhan, China

**Keywords:** artificial intelligence, clinical photography, deep learning, early screening, medical imaging

## Abstract

Oral cancer is a globally prevalent and life-threatening malignancy, where early detection can significantly improve prognosis and reduce mortality. Traditional screening methods are often limited by operator dependence, invasiveness, and high costs, leading to frequent late diagnoses. This systematic review aims to evaluate the current application of artificial intelligence (AI) technology in the early diagnosis and risk prediction of oral cancer, with a focus on diagnostic accuracy, methodological diversity, and clinical translatability. Methods: We conducted a systematic search across five databases (PubMed, Embase, Cochrane Library, Web of Science, and Scopus), incorporating 63 high-quality studies. The analysis was performed at two levels: data input modalities and the evolution of AI algorithms. Study selection, data extraction, and quality assessment followed standard systematic review protocols. Results: AI models demonstrated high sensitivity and specificity in detecting early oral lesions and differentiating precancerous lesions, showing a trend toward multimodal fusion, lightweight, and high-performance development. However, most studies faced challenges such as insufficient sample sizes, limited external validation, and poor model interpretability. Conclusion: AI holds significant potential for improving early oral cancer screening. To fully realize its clinical value, it is essential to establish large-scale multicenter datasets, conduct rigorous prospective validation, enhance model transparency, and address ethical and privacy concerns.

## 1 Introduction

Oral cancer represents a significant global public health challenge (1). Annually, approximately 370,000 new cases are diagnosed and 170,000 deaths result from this disease, with over two-thirds of these cases occurring in Asia (2). Established high-risk factors include tobacco use, alcohol consumption, betel nut chewing, and human papillomavirus (HPV) infection. Notably, the synergistic interaction between betel nut use and tobacco smoking substantially elevates the risk of oral cancer in South and Southeast Asian countries (3). Oral cancer poses a life-threatening risk and significantly compromises patients’ speech, swallowing, and masticatory functions, imposing substantial long-term social and economic burdens (4, 5).

Despite the disease’s high preventability, early detection rates remain suboptimal. Over 60% of patients receive a diagnosis at stages III–IV, at these advanced stages, the 5-year survival rate is typically below 50%. Conversely, for patients diagnosed at stages I–II, the 5-year survival rate can reach 80–90% with timely diagnosis and intervention (6). Consequently, enhancing early screening capabilities represents a critical strategy for reducing mortality and improving prognosis (7). However, traditional screening methods exhibit significant limitations: visual inspection is heavily dependent on physician experience and highly subjective in nature, frequently influenced by training levels and resource availability; biopsy, while the diagnostic gold standard (8), is invasive and associated with high costs, rendering it unsuitable for large-scale population screening; imaging techniques, such as CT and MRI, demonstrate limited sensitivity for detecting early-stage or small lesions and are costly, thereby constraining widespread implementation. These limitations substantially contribute to the current low rate of early diagnosis (9).

With the rapid advancement of artificial intelligence (AI) technology, the field of medical screening is undergoing a revolutionary transformation. The core advantage of AI lies in its ability to simulate human learning, reasoning, and judgment processes through algorithmic computation, thereby effectively identifying potential patterns within complex datasets (10). Traditional diagnostic methods rely on physician experience and limited feature extraction, whereas AI technology can efficiently process large-scale multidimensional data, yielding analysis results that are objective, consistent, and operationally efficient (11). This capability aligns closely with the core challenges of early cancer screening, which include “difficult-to-identify subtle features, massive data volumes, and significant information heterogeneity” (12).

Among the major branches of artificial intelligence, machine learning (ML) stands as the most fundamental and widely applied methodology. Through algorithmic training, machine learning enables tasks such as classification, clustering, and prediction in feature spaces. Its advanced form, deep learning (DL), employs multi-layer neural network architectures to extract hierarchical feature representations, thereby demonstrating unique advantages in processing high-dimensional complex data modalities such as images and sequences. As a primary subset of deep learning, convolutional neural networks (CNNs) have become leading technologies in medical image analysis. These networks can directly learn spatial patterns from raw image data without requiring manual feature engineering (13). Additionally, Transformer-based deep learning architectures exhibit stronger global modeling capabilities in medical text and imaging tasks, offering significant potential for addressing multimodal integration and cross-domain challenges in AI applications. Overall, these technologies represent the primary application directions of artificial intelligence in the medical field.

In the field of oral health research, computer vision (CV) is a key research direction for the application of deep learning methods. CV technology can automatically identify and delineate anatomical structures and pathological abnormalities in medical images, including lesion boundaries, morphological changes, and microscopic texture features. Meanwhile, advancements in digital pathology have enabled comprehensive analysis of tissue sections and cytological specimens through whole-slide imaging systems, thereby promoting the widespread application of artificial intelligence in pathological screening and risk stratification (14). With the continuous development of artificial intelligence technologies, natural language processing (NLP) methods have demonstrated significant potential in medical knowledge extraction and electronic health record (EHR) analysis, providing complementary data sources for multimodal diagnostic frameworks.

The potential of artificial intelligence (AI) in early cancer screening has been extensively validated. For instance, in breast cancer screening, deep learning algorithms have achieved accuracy rates in identifying minute calcifications on mammograms that match or even surpass those of experienced radiologists (15). Similarly, in low-dose CT screening for lung cancer, AI systems can identify early nodules and perform risk stratification through high-dimensional image feature analysis. These technological advancements provide valuable insights for early oral cancer screening. Like breast and lung cancers, early oral cancer lesions often present highly subtle clinical and imaging characteristics, making them prone to missed diagnosis. Therefore, the pattern recognition capabilities of AI are particularly crucial in early oral cancer screening (16). Although AI applications in oral cancer research are gradually advancing, systematic reviews of AI’s role in early oral cancer screening remain lacking. To address this, this study systematically reviews existing literature and comprehensively explores the application value and practical pathways of AI in various types of early oral cancer screening.

## 2 Methodology

### 2.1 Search strategy

This review was conducted in strict compliance with the PRISMA guidelines for systematic reviews and meta-analyses, without violating any ethical or regulatory standards. The core research question of “The Application of Artificial Intelligence in Early Oral Cancer Screening” was identified using the PICOS strategy. The study subjects (P) were artificial intelligence, the intervention (I) involved the use of AI in early oral cancer screening, the control (C) group comprised clinicians or those not utilizing AI, the outcome (O) was quantifiable numerical data, and the study design (S) included *in vivo* or *in vitro* experiments.

In February 2026, we conducted a systematic search across five databases—PubMed, Embase, Web of Science, Cochrane Library, and Scopus—to retrieve studies evaluating the application of artificial intelligence (AI) in dental root canal therapy. The search utilized subject terms and text terms including all peer-level medical subject headings (MESH) related to AI and oral cancer, such as: Mouth Neoplasm, Neoplasm, Mouth, Neoplasms, Oral, Neoplasms, Oral, Neoplasms, Oral, Neoplasms, Oral Neoplasm, Oral Neoplasms, Mouth Cancer, Mouth Cancers, Mouth Cancer, Cancer, Mouth, Cancers, Mouth, Oral Cancer, Cancer, Oral, Cancers, Oral, Oral Cancers, Mouth Cancer, and Intelligence, Artificial, Computer Reasoning, Reasoning. Computer, AI (Artificial Intelligence), Machine Intelligence, Intelligence, Machine, Computational Intelligence, Intelligence, Computational, Computer Vision Systems, Computer Vision System, System, Computer Vision, Systems, Computer Vision, Vision System, Computer, Vision Systems, Computer, Knowledge Acquisition (Computer), Acquisition, Knowledge (Computer), Knowledge Representation (Computer), Knowledge Representations (Computer), Representation, Knowledge (Computer).

### 2.2 Inclusion criteria and exclusion criteria

The inclusion criteria for this systematic review are as follows: (1) Published peer-reviewed studies; (2) Research articles with original and quantifiable experimental conclusions; (3) Main content related to artificial intelligence and early screening of oral cancer; (4) Studies exploring the application of AI methods in early screening of oral cancer; (5) Algorithms with results validated by internal or external reports; (6) Studies published exclusively in English.

The exclusion criteria were as follows: (1) non-study articles, (2) articles not published in English, (3) articles with themes inconsistent with early oral cancer screening or artificial intelligence, (4) publications from non-academic sources such as newspapers, journals, and blogs, (5) articles lacking original quantifiable experimental results, (6) studies unrelated to humans, (7) case studies, and editorial reports.

### 2.3 Article selection

**Figure 1:**
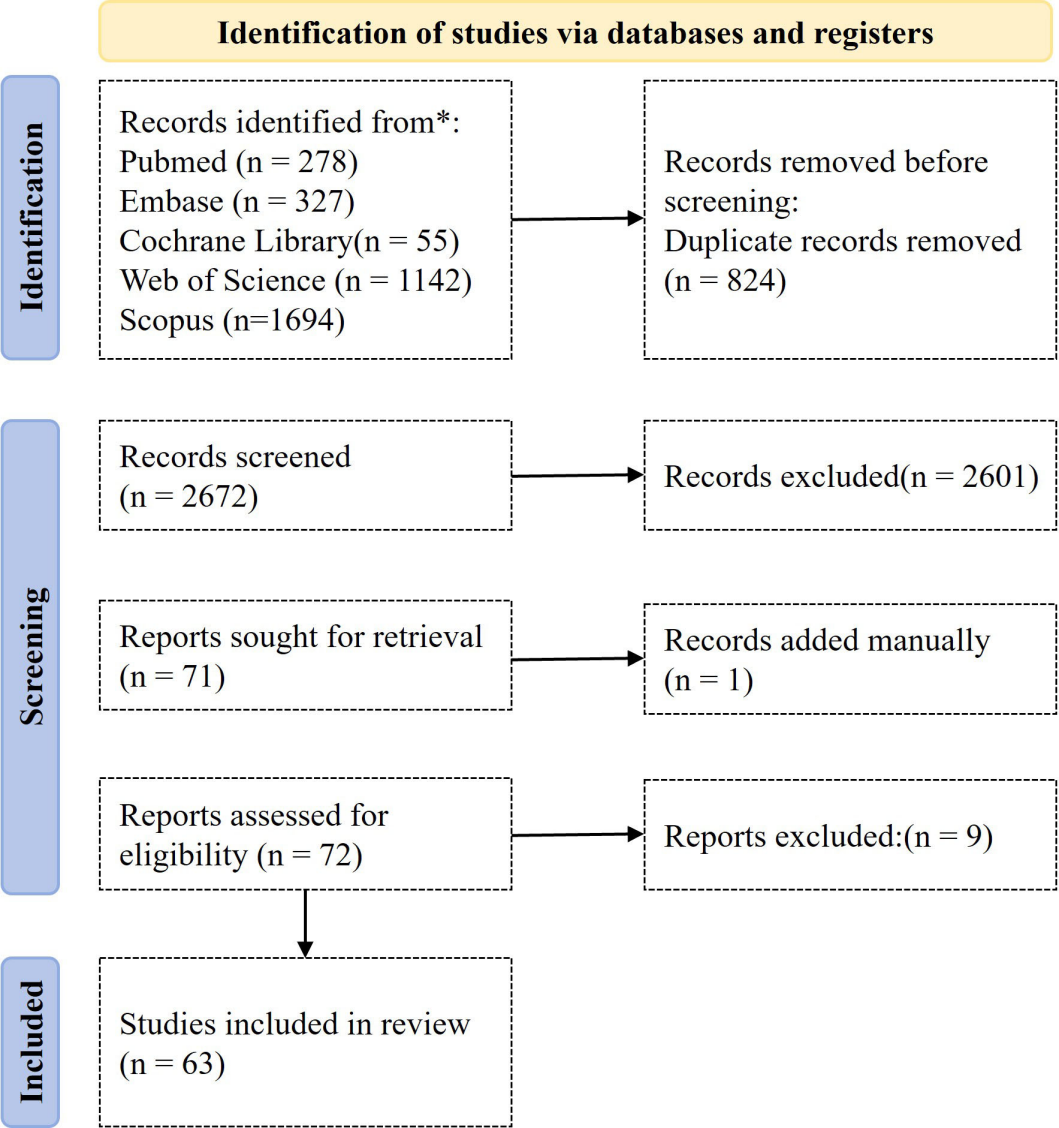
PRISMA flowchart showing the systematic selection of article.

A total of 3,496 articles were identified based on the search criteria. After preliminary screening to exclude 824 duplicate or similar entries, 2,672 candidate papers were selected. Following title and abstract reviews, 2,601 were further eliminated. Full-text retrieval was completed for the remaining 71 papers, with an additional manual review of 1 reference. Ultimately, 72 papers were approved for full-text access, among which 9 were excluded due to non-compliance with quality standards. After comprehensive evaluation, 63 papers were finally selected for inclusion in the study (**Figure 1**).

## 3 Application of AI in early oral cancer screening

This section elaborates on various input data modalities for early screening of oral cancer, including optical and radiographic imaging, pathological imaging, molecular and cellular imaging, as well as clinical data and prognostic modeling. The following provides an in-depth analysis of each moda (**Table 1**).

**Table 1:** Important features of studies included in the systematic review.

Author with year	Objective	Modality	Results	Conclusion
Pushparathi VPG et al, 2025 (17)	Develop DL model for OC classification to enhance diagnostic decision-making and interpretability.	Vahadane normalisation + WFS + Improved U-Net + XAI; Histopathology images.	Accuracy 99.54%; Outperforms DenseNet201/VGG10 in precision/reliability.	Suitable for early precise OC detection; reduces diagnostic errors via explainability.
Bashir RMS et al, 2023 (18)	Develop prognostic models for malignant transformation in OED using histology WSIs.	Weakly supervised DL; OED tissue sections (n=137 patients).	AUROC 0.78 (5-fold CV); PELs count & nuclei count significant (p<0.05).	First application of DL for OED prognostication; offers potential to aid patient management.
Guedes IC et al, 2025 (19)	Compare effectiveness of human analyses versus AI system assessment of oral cell smears (Pilot Study).	AI system (PSIE) vs. Human; Pap-stained smears (57 patients).	Concordance good; PSIE analysis time 16.6x shorter than human.	AI promising for OSCC screening; suitable for routine use to accelerate analysis.
Warin K et al, 2022 (20)	Evaluate CNN algorithms to classify and detect OPMDs in oral photographs.	DenseNet-121, ResNet-50, Faster R-CNN; 600 oral photographs.	Classification AUC 95% (Sen 100%, Spe 90%); Detection AUC 74.34%.	Models have potential for classification and detection of OPMDs in oral photographs.
Warin K et al, 2021 (21)	Develop automated classification and detection model for oral cancer screening.	DenseNet121, Faster R-CNN; 700 clinical oral photographs.	DenseNet121: Acc 99%, Sen 98.75%, Spe 100%, AUC 99%.	Algorithms offer acceptable potential for classification and detection of cancerous lesions.
Desai KM et al, 2025 (22)	Evaluate effectiveness of AI screening tools for OPMDs/Oral cancers via smartphone devices.	DenseNet201, FixCaps; 518 oral cavity images.	DenseNet201: F1 87.50%, AUC 0.97, Acc 88.6%. FixCaps: Acc 83.8% (0.83M params).	DenseNet201 robust for cloud; FixCaps lightweight for native phone-based screening.
Yuan W et al, 2022 (23)	Propose novel deep learning method for noninvasive oral cancer screening on OCT images.	LRAN (RFR + LDA modules); OCT images (25 patients).	Accuracy 91.62%, Sen 91.66%, Spe 92.58%.	LRAN model demonstrates excellent capability to solve noninvasive screening tasks.
Yuan W et al, 2023 (24)	Develop automatic noninvasive OSCC diagnosis approach on OCT images.	MDRL network; 460 OCT images (37 patients).	Image-level: Sen 91.2%, Spe 83.6%, Acc 87.5%, AUC 0.92.	Developed DL system expresses superior performance vs. traditional CNNs and specialists.
Ahmad M et al, 2023 (25)	Develop hybrid methodologies based on fused features for early diagnosis of OSCC.	Transfer Learning + SVM + Hybrid Feature Fusion (CNN+GLCM/HOG/LBP); Histology.	DenseNet201+Hybrid+SVM: Acc 97.00%, Sen 90.90%, Spe 98.92%, AUC 96.80%.	Proposed system achieved promising results for rapid diagnosis using histological images.
Liyanage V et al, 2023 (26)	Explore utility/feasibility of smartphone app to photograph and diagnose oral lesions (Proof-of-concept).	MobileNetV3, EfficientNetV2; 342 images (3 categories).	EfficientNetV2: Val Acc 0.71 (Premalignant/Malignant 80%).	AI software has potential accuracy in distinguishing malignant lesions for remote screenings.
Bur AM et al, 2019 (27)	Develop/validate algorithm to predict occult nodal metastasis in clinically node negative OCSCC.	Machine Learning (Decision Forest); NCDB data (782 patients).	Decision Forest AUC = 0.840; Exceeded DOI model (AUC = 0.657, p=0.007).	ML improves prediction of pathologic nodal metastasis compared to DOI methods.
Alanazi AA et al, 2022 (28)	Develop IDL-OSCDC technique using biomedical images.	Gabor filtering + NasNet + EGOA-based DBN; Benchmark dataset.	Accuracy 95%, Precision 96.15%, Recall 93.75%, F1-score 94.67%.	IDL-OSCDC model highlights promising performance over other methods.
Tafala I et al, 2025 (29)	Present DeepPatchNet for histopathological image classification.	DeepPatchNet (DeepLabV3+ + ConvMixer); NDB-UFES (3763 images).	Accuracy 86.71%, Precision 86.80%, Recall 86.71%, F1-score 86.75%.	Shows strong potential as reliable, lightweight, interpretable decision support tool.
Akram K et al, 2026 (30)	Introduce Deep Visual Detection System (DVDS) to automate OSCC detection.	EfficientNetB3, DenseNet121, ResNet50; Kaggle (5192 imgs) + NDB-UFES (3763 imgs).	EfficientNetB3: Binary Acc 97.05%, Spe 97.17%, Sen 96.92%.	DVDS demonstrates high reliability/robustness for deployment in clinical settings.
Lin Z et al, 2025 (31)	Apply EIS with deep learning to assist clinical diagnosis of OPMD/oral cancer.	EIS + CNN; Porcine mucosa + Human (51 healthy, 11 patients).	Human Binary: AUC 0.91, Acc 0.91, Spe 0.97, Sen 0.74.	EIS combined with CNN models demonstrates considerable potential as adjunctive non-invasive tool.
Lepper TW et al, 2025 (32)	Define AgNOR cut-off risk points by oral exfoliative cytological smears.	AgNOR Slide-Image Examiner (CNN); 4 experimental groups.	Cut-off 3.69 AgNORs/nucleus: Sen 86%, Spe 93%, Acc 90%. CNN time 20 min vs. human 67h.	Cut-off point indicates suspicious sample; contributes to improvements in screening.
Xie X-M et al, 2023 (33)	Demonstrate accurate diagnosis of OC using AI-based SERS of exhaled breath.	Ag NWs@ZIF nanochains + SERS + ANN; 400 spectra.	ANN Accuracy 99%; AUC 0.996 for simulated OC breath.	Combination of breath analysis and AI shows great potential for early-stage diagnosis.
Gürses BO et al, 2025 (34)	Evaluate diagnostic potential of surface texture features in differentiating benign/malignant lesions.	Surface texture features + SVM; 275 intraoral photographs.	SVM: Sen 99.2%, Spe 81.4%, Acc 90.5%, AUC 0.939.	Surface texture features offer promising diagnostic indicators; SVM demonstrated robust performance.
Fatapour Y et al, 2023 (35)	Develop framework to generate ML prediction models for OTSCC recurrence.	SEER database + H2O.ai (4 models); 130,979 patients.	Gradient Boosting: 5-year Acc 81.8%, Precision 97.7%. 10-year Acc 80.0%.	Novel SEER framework enabled successful identification of patients with OTSCC recurrence.
Kouketsu A et al, 2024 (36)	Construct AI-based model for detecting oral cancer/dysplastic leukoplakia using oral cavity images.	Single Shot Multibox Detector; 1043 images (523 training).	OSCC detection: Sen 93.9%, NPV 98.8%, Spe 81.2%.	Proposed model is a potential diagnostic tool for oral diseases.
Warin K et al, 2022 (4)	Evaluate performance of deep CNN algorithms for classification/detection of OPMDs/OSCC.	DenseNet-169, etc. (Class); Faster R-CNN, etc. (Det); 980 images.	DenseNet-196 Class AUC: OSCC 1.00, OPMDs 0.98. Det AUC: OSCC 0.88.	CNN-based models have potential for identification; expected to assist GPs for early detection.
Talwar V et al, 2023 (37)	Evaluate utility of AI-based techniques for detecting OPMDs in Indian population using smartphone images.	DenseNets, Swin Transformer; 1120 suspicious + 1058 non-suspicious images.	Internal Test: DenseNet201 F1 0.84. Set I (untrained): F1 0.73.	Proposed AI model has potential to identify suspicious/non-suspicious lesions using photos.
Jubair F et al, 2022 (3)	Develop lightweight deep CNN for binary classification of oral lesions.	EfficientNet-B0 (lightweight); 716 clinical images.	Accuracy 85.0% (95% CI: 81.0%-90.0%), AUC 0.928.	Deep CNNs effective for low-budget embedded vision devices; improves screening reach.
Keser G et al, 2024 (38)	Evaluate performance of diagnostic software to detect oral cancer lesions in intra-oral images.	Cranio Catch + YOLOv5; 65 anonymous retrospective images.	Test images: F1, Sen, Precision all 0.667.	AI shows promise in prediagnosis; success rates will increase with more images.
de Chauveron J et al, 2026 (39)	Evaluate potential of computer vision models as diagnostic tools for identifying cancerous lesions.	Modern DL object detection models; Biopsy-proven dataset.	High performance in single-class detection; challenging in two-class (malig vs benign).	Underscores AI potential while identifying areas for improvement (small lesion detection).
Wuttisarnwattana P et al, 2024 (40)	Demonstrate use of DL-based AI for simultaneously identifying types/outlining boundary of lesions.	DL-based AI for segmentation; OPMDs and OSCC lesions.	Model successfully (1): detected lesions (2), determined types (3), outlined boundaries.	Patients will be diagnosed/treated early before pre-cancer lesions progress.
Rahman T et al, 2019 (41)	Extract shape, texture, color features from histopathological images for automated OSCC identification.	42 whole slide slices (720 nuclei); Decision Tree, SVM, etc.	Shape (Tree): 99.4% acc. Texture/Color (SVM/Logistic): 100% acc.	Achieved result can be effectively converted to software as assistant diagnostic tool.
Adeoye J et al, 2024 (42)	Investigate whether DL model can predict dysplasia probability among leukoplakia patients using photos.	EfficientNet-B2; 2,073 retrospective images; Temporal/Geo validation.	Testing: AUC 0.882, Balanced Acc 81.8%. External: AUC 0.828, Acc 76.4%.	Model shows potential for decision support to select patients for biopsy/obviate unnecessary biopsy.
Keser G et al, 2025 (43)	Evaluate performance of DL-based diagnostic software designed to detect OPMD (Retrospective Pilot).	YOLOv8; 358 anonymized retrospective intraoral images.	F1 score 0.693, Sen 0.666, Precision 0.723.	DL/AI show promise in OPMD diagnosis; larger datasets needed for clinical translation.
Saldivia-Siracusa C et al, 2025 (44)	Develop/assess AI models for automatic classification of OPMD/OSCC clinical images (Cross-sectional).	8 CNNs (Transfer Learning); 778 clinical images (8:1:1 split).	ConvNeXt/MobileNet best. Acc 0.799, Precision 0.837, AUROC 0.863.	Adoption of DL models in healthcare could aid in diagnostic assistance/decision-making.
Kabir MF et al, 2025 (45)	Propose advanced DL-based diagnostic model, LightSE-MobileViT, to classify OC.	LightSE-MobileViT; 131 original images (augmented to 981).	Accuracy 98.39%, Precision/Recall ~1.00, Macro F1 0.98, ROC-AUC 1.00.	Exceptional performance underscores robust capability; significant potential for automated screening.
Xu S et al, 2019 (46)	Establish 3DCNNs-based image processing algorithm for early diagnosis of oral cancers.	3D/2D CNNs; CT images with dynamic enhancement rate.	3DCNNs with dynamic enhancement performed better than 2DCNNs.	Spatial features/dynamics from 3DCNNs may inform future design of CT-assisted diagnosis.
Musulin J et al, 2021 (47)	Propose two-stage AI-based system for automatic multiclass grading/segmentation of tissue.	Xception + SWT (Class); DeepLabv3+ (Seg).	Class: AUCmacro 0.963. Seg: mIOU 0.878, F1 score 0.955.	Proposed AI-based system has great potential in OSCC diagnosis; reduces variability.
Babu PA et al, 2024 (48)	Present unique approach to early detection/diagnosis of OC using deep neural networks.	Deep neural networks; Transfer learning; Inception-V3.	Inception-V3 exhibited superior accuracy compared to other algorithms.	DL effectively addresses this challenging problem; regular dental exams vital.
Welikala RA et al, 2020 (49)	Assess two DL based computer vision approaches for automated detection/classification of oral lesions.	ResNet-101 (Class); Faster R-CNN (Det); MeMoSA^®^ project data.	Class F1: 87.07% (lesions), 78.30% (referral). Det F1: 41.18% (referral).	Initial results demonstrate DL has potential; building large annotated library is key.
Ananthakrishnan B et al, 2023 (50)	Classify normal/carcinogenic cells in oral cavity using two different approaches.	Approach 1: LBP+Histogram+ML. Approach 2: CNN feature + Random Forest.	400× imgs: Acc 96.94%, AUC 0.976. 100× imgs: Acc 99.65%, AUC 0.9983.	Proposed work produces high test accuracy; bypasses large data requirement problem.
Lin H et al, 2021 (51)	Present effective smartphone-based imaging diagnosis method powered by DL.	Centered rule image-capturing + Resampling + HRNet; 455 test images.	Sen 83.0%, Spe 96.6%, Precision 84.3%, F1 83.6%.	Smartphone-based imaging with DL method has good potential for primary OC diagnosis.
Ohba S et al, 2023 (52)	Investigate two methods for automatic detection of cell nuclei in oral cytology.	SWM vs. Mask-RCNN; 30 cases of liquid-based oral cytology.	SWM rate 0.9314. Mask-RCNN error rate 0.027 (more accurate).	Mask-RCNN more accurate in identifying cell nuclei; improves diagnostic performance.
Goswami B et al, 2024 (53)	Propose method to differentiate benign/malignant lesions and classify pre-cancerous stages.	5 color spaces + LightGBM; White Light Images.	Binary: Acc 99.25%, Precision 99.18%, Spe 99.31%. Multi-class: Acc 98.88%.	Proposed method uses hand-crafted features/ML; promising performance with limited resources.
Jeyaraj PR et al, 2022 (54)	Present infrastructure approach to detect/classify OC from hyperspectral imaging.	DBM + SVM fusion; Hyperspectral imaging.	Classifier accuracy 94.75% by classifier fusion.	Proposed digital pre-screening framework provides high potential cancer identification tool.
Chang X et al, 2023 (55)	Test/compare/analyze performance of classical DNN models recognizing OC tissues using Raman spectroscopy.	AlexNet, VGGNet, ResNet50, etc.; 16,200 Raman spectra (90 patients).	ResNet50 best: Acc 92.81%, Precision 92.93%, Recall 92.86%.	Work provides guide for OC detection using DL with Raman spectroscopy.
Marzouk R et al, 2022 (56)	Introduce AI with Deep Transfer Learning driven OC detection and Classification Model (AIDTL-OCCM).	Fuzzy-based contrast + DenseNet-169 + COA + AE.	Maximum accuracy of 90.08%; enhanced performance compared to other approaches.	Extensive experimental analysis established enhanced performance of AIDTL-OCCM model.
Chen Z et al, 2024 (57)	Introduce efficient/rapid approach for mass determination of OC differentiation levels.	Serum Raman spectroscopy + DNN + XGBoost.	(Abstract emphasizes method efficiency/reliability).	Achieved most accurate/reliable classification of OC differentiation stages.
Fati SM et al, 2022 (58)	Achieve satisfactory results for early diagnosis of OSCC by applying hybrid techniques.	Method 1: CNN+SVM. Method 2: CNN features + Handcrafted + PCA + ANN.	ANN (Hybrid): Acc 99.1%, Spe 99.61%, Sen 99.5%, Precision 99.71%, AUC 99.52%.	All proposed systems achieved superior results; hybrid feature fusion significantly improves diagnosis.
Zafar A et al, 2024 (59)	Present automatic image-processing-based ML approach for OSCC detection.	Deep feature fusion (ResNet-101 + EfficientNet-b0) + b-IHHO; Histopathology.	Classification rate 97.78%; b-IHHO trained k-NN with avg feature vector size 899.	Proposed framework may be applicable in clinical settings to aid doctors.
Guo Z et al, 2024 (60)	Propose OC diagnosis approach based on few-shot learning framework.	Prototypical network; Dual feature extractors; Histopathological images.	(Abstract states superior performance over comparison methods).	Proposed approach demonstrates superior performance; addresses data scarcity challenge.
Rabinovici-Cohen S et al, 2024 (61)	Investigate use of clinical photographic images taken by smartphones for automated detection of OSCC.	Various DL methods; Retrospective study (1470 patients).	Holdout: AUC 0.96, Sen 0.91, Spe 0.81. Lesion location stratified: AUC 1.00.	Results underscore potential of leveraging clinical photos for timely/accurate identification.
Awais M et al, 2020 (62)	Focus on HPIL system to support diagnosis via advanced machine learning.	Autofluorescence + GLCM texture + LDA + KNN.	Accuracy 83%, Sen 85%, Spe 84% in differentiating standard/anomalous regions.	Method may help dental specialist identify anomalous regions for biopsies more efficiently.
Monani UJ et al, 2025 (63)	Propose reliable ML system for OC detection using CNNs and transfer learning.	CNNs + Transfer Learning; FC1 + FC2; SMOTE + Data Augmentation.	Test: F1-score 81.48%, Acc 81.38%, ROC-AUC 0.9082.	Highlights how AI can impact clinical workflows; results offer hopeful path for advancements.
Pham TD et al, 2025 (64)	Introduce approach to classify histopathological images for detecting dysplasia via fusion of SVM classifiers.	InceptionResNet-v2 + SVM; ViT + SVM; Fusion strategy.	Fusion approach significantly outperformed individual models; superior balanced accuracy/AUC.	Highlights potential of integrating DL feature extraction with SVM classifiers.
Chaudhuri D et al, 2023 (65)	Utilize MLA ensembles for early OC risk assessment using Raman cyto-spectroscopy.	2 MLA ensembles (1): LDA+DTC (2), LDA+SVM; Oral epithelial cells RS.	Ensembles Acc (88 ± 8%) > DTC (48 ± 19%) or SVM (58 ± 15%).	LDA-SVM ensemble can be used to accurately detect OC risk and screen susceptible patients.
Afify HM et al, 2023 (66)	Propose multimodal deep-learning pipeline incorporating diverse data sources.	6 pre-trained DL models; Image encoders + Patient metadata.	MobileNetV3-Large: Acc 81%, Precision 79%, Recall 79%, F1-score 78%, MCC 0.57.	Findings highlight efficacy of integrating multiple data modalities for more accurate detection.
Yu HSU et al, 2024 (67)	Leverage computer vision/DL to enhance early detection/classification of oral mucosal lesions.	YOLOv7; 6903 images expanded to >50,000; Three referral grades.	YOLOv7-E6 high precision/recall. YOLOv7-D6 excelled at malignant lesions.	Results highlight potential of DL models to contribute to early detection/remote screening.
Xie F et al, 2024 (68)	Develop DSDF model for identifying oral mucosal diseases.	DSDF model: Dynamic self-attention + Feature discrimination loss.	Accuracy 91.16% (~6% ahead of other methods); Recall 90.87%, F1 90.60%.	DSDF extracts distinguished visual features better; conducive to auxiliary diagnosis.
Yang SM et al, 2023 (69)	Evaluate screening accuracy in tobacco users vs. non-users of prototype smartphone/ML algorithm.	Prototype platform; AFI + pWLI; 318 subjects (Smokers/Non-smokers).	Smokers: Sen 80.0%, Spe 87.5%, Agreement 83.67%. Non-smokers: Sen 62.1%, Spe 82.9%.	Tobacco use should be carefully weighted as variable in architecture of imaging-based screening.
McRae MP et al, 2020 (70)	Describe cytopathology tools (ML algorithms, clinical algorithms) to assist pathologists/clinicians.	Cytology-on-a-chip; ML model (4 cell phenotypes); 999 subjects.	Cell phenotypes accuracy 99.3%. Clinical algorithms: AUC 0.81 (benign vs mild), 0.95 (benign vs malig).	New cytopathology tools represent practical solution for rapid PMOL assessment.
Adeoye J et al, 2023 (71)	Compare/select optimal ML-based models for stratifying malignant transformation status.	46 ML-based models; 1,187 patients (3 institutions); 26/15 predictors.	26 predictors: Acc 97%. 15 predictors: Acc 94%. External Val: Sen 1, Spe 0.88, F1 0.67.	ML-based models could be useful to stratify patients according to risk of malignant transformation.
Confer MP et al, 2024 (72)	Employ QCL-based DFIR chemical imaging to record data from oral tissues for tissue segmentation.	QCL-based DFIR + CNN; 3 bands + darkfield visible imaging.	94.5% accurate with ground truth (pathologist-annotated H&E).	Chemical-imaging-based workflow for OPMD classification has potential to enhance efficiency.
Nemichand et al, 2025 (73)	Present smartphone-based bimodal device (SBBD) combining fluorescence imaging/spectroscopy.	SBBD (405 nm laser); 2D CNN + ANN; 136 subjects.	2D CNN: Acc 97.04%, Sen 96.13%, Spe 97.73%. ANN Spectroscopy: Acc 97.44%.	Bimodal approach effectively addresses diagnostic gap occurring when spectroscopy/imaging used independently.
Rahman TY et al, 2020 (74)	Identify OSCC based on morphological/textural features of hand-cropped cell nuclei.	40 biopsy slides (452 nuclei); 5 classifiers (SVM, Logistic, etc.).	Decision Tree classifier: 99.78% accuracy using morphological/textural features.	Morphological/textural features play very important role in OSCC diagnosis.
Zayed SO et al, 2024 (75)	Develop software with all needed feeding data to act as AI-based program to diagnose oral diseases.	DODS program; 28 diseases; 11,200 texts/3000 images.	Correct diagnosis rates: Group 1 (Software) 87%, Group 2 (Microscopic) 90.6%, Group 3 (Hybrid) 95%.	DODS correct diagnosis rate comparable to oral pathologists; could be utilized as diagnostic guidance tool.
Parola M et al, 2024 (76)	Propose solution composed of Case-Based Reasoning and Informed Deep Learning (IDL) for screening.	Case-Based Reasoning + IDL; Ensemble architecture.	IDL Accuracy 85% (surpassing DL alone 77%); IDL generates explanations more congruent with clinical users.	Solution lies in capability to handle data imperfections; IDL approach yields higher accuracy/explainability.
Uthoff RD et al,2018 (77)	Development of a low-cost, portable, smartphone-based dual-modal (self-illuminated + polarized white light) dual-view imaging system for oral cancer screening in resource-limited communities.	An automatic classification algorithm based on CNN for DL.	Sensitivity and specificity metrics ranging from 81% to 95% in distinguishing benign from malignant lesions; the screening accuracy for information analysis to 86.6%.	Significantly enhance the accuracy of oral cancer screening, providing a feasible technical solution for implementing efficient and cost-effective screening in regions with inadequate infrastructure.

### 3.1 Advanced imaging modalities

Early lesions of oral cancer typically manifest as mild leukoplakia, erythematous lesions, or superficial ulcers, which are difficult to assess visually. Diagnosis often requires the use of advanced imaging techniques, including smartphone photographs, clinical SLR photographs, optical coherence tomography (OCT), autofluorescence imaging, hyperspectral imaging, and electrical impedance spectroscopy (EIS). The advantages of advanced imaging lie in their non-invasiveness, real-time capability, and accessibility, making them primarily applicable for large-scale population screening and triage.

In the field of visible light photo screening, models based on CNNs have demonstrated diagnostic performance approaching expert-level accuracy. A series of studies by Warin et al. (4, 20, 21) confirmed that DenseNet and Faster R-CNN models achieved AUC values of 95%-99% and sensitivity up to 100% in oral photo classification, significantly outperforming general practitioners. The Uthoff team developed a bedside dual-modal imaging system based on convolutional neural networks for smartphone image classification, achieving a sensitivity of 92.59% and specificity of 86.67% in the detection of oral cancer and precancerous lesions, demonstrating the feasibility of AI-assisted analysis of non-clinical images for early screening of oral cancer (77). To enhance accessibility in primary healthcare, research trends are shifting from cloud processing to edge computing. Desai et al. (22) compared cloud-based models (DenseNet201) with lightweight local models (FixCaps, 0.83 million parameters), which maintained an accuracy rate of 83.8% while being more suitable for mobile device deployment. The LightSE-MobileViT model proposed by Kabir et al. (45) achieved an accuracy rate of 98.39% and ROC-AUC of 1.00 on clinical validation datasets, further demonstrating the potential of lightweight Transformer architectures in automated screening. However, image quality variability remains a major bottleneck. Talwar et al. (37) found that when test set images were captured by untrained personnel, the model’s F1 score dropped from 0.84 to 0.73, suggesting the need for standardized image acquisition procedures (e.g., the centering method proposed by Lin et al. (51)).

Based on clinical photographs, AI models also demonstrated quantitative diagnostic capabilities. Lin et al. established a standardized image acquisition and training protocol emphasizing image centering and resampling. When evaluated on a multi-class test set comprising five different types of oral lesions, the model achieved 83.0% sensitivity and 96.6% specificity (32). Large-scale studies utilizing clinical images also yielded consistent results. A cascade CNN AI model developed using 44,409 multicenter clinical photographs for automated oral cancer detection achieved high overall discriminative performance on an independent validation set (internal AUC = 0.983; external AUC = 0.935) (33). For subtle lesions difficult to identify by the naked eye (including early leukoplakia and erythroplakia), Camalan et al. employed CNN to analyze clinical images of potential oral malignancies. By combining end-to-end classification with interpretable heatmaps to highlight diagnostic regions, the model consistently identified visual indicators associated with superficial lesions, providing interpretable guidance for clinical evaluation (34).

A DL model utilizing the MC3 network based on OCT for detecting inflammatory factors in oral cancer demonstrated high accuracy, precision, and reproducibility in preoperative evaluation, prognostic analysis, and outcome prediction, highlighting its potential as a reliable tool for assessing inflammatory risk in oral health diagnostics (29). Additionally, a CNN AI model based on hyperspectral imaging achieved accuracy, sensitivity, and specificity exceeding 90% in classifying benign and malignant oral lesions, significantly advancing the early screening capabilities for oral cancer lesions (30).

Beyond visible light, functional imaging technologies aim to overcome the limitations of morphological information. Yuan et al. (23, 24) demonstrated non-invasive diagnosis using OCT combined with Local Residual Adaptive Networks (LRAN) and Multi-layer Deep Residual Learning (MDRL), achieving an accuracy rate exceeding 91% and a sensitivity of 100% (at patch level), proving the potential of OCT as a substitute for invasive biopsy. Awais et al. (62) explored autofluorescence imaging, which exhibited high sensitivity (96.8%) but low specificity (48.4%), requiring optimization with texture analysis. Lin et al. (31) pioneered the integration of Electrical Impedance Spectroscopy (EIS) with CNN, utilizing differences in tissue electrical properties to distinguish normal from pathological tissues, achieving a specificity of 0.97 and providing a novel physical dimension for non-invasive detection. Jeyaraj et al. conducted research on hyperspectral imaging combined with 3D spatiotemporal CNN (54), demonstrating *in vivo* detection accuracy of 81%-94.75%, highlighting the high specificity of optical biopsy.

Radiographic imaging remains the established diagnostic modality for oral cancer, yet its utility in early detection remains limited. The integration of radiographic imaging with AI models demonstrates significant potential for enhancing early diagnosis of oral cancer. After training an optimized deep learning model on over 4,000 dental X-rays, lesion detection accuracy surpassed that of primary dentists and improved inter-observer agreement (4). Furthermore, DL applied to MRI for head and neck tumor segmentation enables more precise delineation of lesion boundaries, providing valuable assistance to clinical radiologists (37). These findings indicate that AI has the potential to reduce diagnostic omissions in radiographic interpretation and improve the detection rate of subtle lesions. Although current research in this area remains limited, radiographic-based AI models for early oral cancer screening are expected to see broader application in the future.

In general, macroscopic optical imaging is suitable for initial screening but is limited by environmental factors such as lighting and angle; OCT and hyperspectral imaging provide deeper tissue information but are associated with higher equipment costs; CT image analysis is primarily used for advanced staging rather than early screening (46). Through optical imaging, these models can comprehensively evaluate lesion characteristics and related risk factors, thereby assisting clinicians in evidence-based decision-making and predicting surgical outcomes. Expanding sample databases can enhance the accuracy of AI models and reduce the need for repeat examinations. Future trends include multimodal fusion (e.g., dual-mode devices combining fluorescence and spectral imaging by Nemichand et al., 2025) to address the diagnostic gaps of single modalities (73).

### 3.2 Microscopic pathological imaging

Pathological imaging is a critical component of early screening for oral cancer, primarily based on whole slide imaging (WSI) of histopathology, biopsy sections, and cytological smears. As an auxiliary tool to the gold standard of diagnosis, this modality focuses on microscopic analysis of nuclear morphology, histological structure, and dysplasia, aiming to enhance the consistency and efficiency of pathological diagnosis while reducing interobserver variability.

In alignment with screening objectives, recent studies have explored AI applications for risk stratification of precancerous lesions using histological WSIs. For instance, Bashir et al. (18) developed a weakly supervised deep learning model to predict malignant transformation in OED tissue sections, achieving an AUROC of 0.78. This approach demonstrates the potential of AI to identify high-risk dysplasia that warrants closer monitoring or intervention, rather than merely classifying confirmed cancers. Such prognostic tools complement diagnostic models by focusing on lesion evolution instead of static classification.

In the field of histopathological image analysis, most studies have reported extremely high diagnostic accuracy. Pushparathi et al. combined Vahadane normalization with improved U-Net, achieving a classification accuracy of 99.54%, and introduced explainable AI (XAI) to enhance clinical trust (17). The deep vision detection system (DVDS) proposed by Akram et al., based on EfficientNetB3, achieved an accuracy of 97.16% in multi-classification tasks (30). To address tumor heterogeneity, Musulin et al. (47) proposed a two-stage AI system, enabling automatic multi-class classification of epithelial-mesenchymal tissue segmentation (mIOU 0.878). Feature fusion strategies have also been widely adopted; Ahmad et al. (25) and Fati et al. (60) improved the AUC to 96.80%-99.52% by integrating CNN deep features with manual features such as GLCM and LBP. Zafar et al. (61) utilized an improved Haris Hawks optimization algorithm to eliminate redundant features, achieving a classification rate of 97.78%.

As a minimally invasive screening method, AI is primarily employed to accelerate nuclear identification and quantification in cytology. Guedes et al. (19) and Lepper et al. (32) validated the efficacy of AI systems in Papanicolaou smear and AgNOR quantification, demonstrating a 16.6 to 100-fold reduction in analysis time compared to manual methods, with good consistency (ICC = 0.896). The “Cell on Chip” tool developed by McRae et al. (70) achieved an AUC of 0.95 in distinguishing benign from malignant cells by quantifying cell phenotype distribution, showcasing the potential for point-of-care testing (POCT). Ohba (52) compared the sliding window method with Mask-RCNN, the latter exhibiting a nuclear recognition error rate of only 0.027.

Notably, this field is evolving from pure classification to interpretable and small-sample learning. Models of Tafala et al. (29) and Afify et al. (66) integrate Grad-CAM, visualizing heatmaps to highlight diagnostic features. Guo et al. (60) proposed a dual-feature extractor prototype network, leveraging a small-sample learning framework to circumvent the need for extensive training data. Pham et al. (64) focused on predicting malignant transformation of oral epithelial dysplasia (OED), achieving an AUROC of approximately 0.73-0.78, thereby extending from diagnosis to risk stratification.

### 3.3 Molecular & cellular imaging

Molecular and cellular imaging represents a cutting-edge direction in non-invasive molecular diagnostics. This modality does not rely on macroscopic morphology or tissue architecture but identifies tissues by detecting molecular fingerprints, volatile markers, or microscopic nuclear features, exhibiting extremely high specificity. It is primarily applied in early risk assessment.

Raman spectroscopy and surface-enhanced Raman spectroscopy (SERS) are core technologies in this field. Chang et al. (55) and Chen et al. (57) utilized Raman spectroscopy combined with ResNet50 and extreme deep learning models to achieve an accuracy rate of 92.81% in identifying oral cancer tissues and differentiation levels. Xie et al. (33) innovatively integrated SERS with exhaled breath analysis, detecting volatile biomarkers (e.g., methyl mercaptan) with an AUC as high as 0.996, providing a novel paradigm for ultra-early non-invasive screening. Chaudhuri et al. (67) employed Raman cell spectroscopy combined with machine learning algorithm integration (LDA-SVM) to achieve an accuracy rate superior to single models (88 ± 8%) in cancer risk prediction, indicating its applicability for screening susceptible patients.

At the genomic level, common mutations in OSCC include TP53, NOTCH1, and PIK3CA. These genetic alterations are critically implicated in tumor initiation, progression, and therapeutic resistance (39). Utilizing high-throughput sequencing data of these genes, machine learning and deep learning models can systematically analyze thousands of genetic features to identify key oncogenic drivers, thereby facilitating the differentiation between patients and healthy individuals. Studies have demonstrated that algorithms such as random forests and support vector machines are effective in constructing classifiers, achieving diagnostic accuracy of 80%–90% in distinguishing malignant from non-malignant samples. This highlights the unique advantages of artificial intelligence in processing complex omics datasets and discovering potential early biomarkers (including the detection of malignant cases before lesion formation) (40).

At the transcriptomic level, miRNAs have garnered significant attention due to their stability in blood and saliva (41). Extensive evidence indicates that alterations in miRNA expression reflect the progression from precancerous lesions to cancer (42). By modeling miRNA expression profiles using deep neural networks or machine learning algorithms, it is possible to effectively distinguish healthy controls, individuals with oral potential malignant lesions (OPML), and early-stage OSCC patients. Certain models have demonstrated high diagnostic accuracy, with AUC values exceeding 0.90 (43). AI screening models utilizing transcriptomic biomarkers provide a promising non-invasive detection method for early oral cancer screening through analysis of saliva or blood samples, thereby avoiding invasive biopsy procedures (44).

Exosomes, as another category of non-invasive biomarkers, have gained increasing recognition for their diagnostic potential (45). Proteins, mRNA, and miRNA encapsulated within exosomes have been implicated in tumor progression (46). Studies have demonstrated that deep learning methods can extract cancer-related features from exosome multi-omics data, exhibiting high accuracy in identifying early-stage cancer patients. Particularly when combined with easily collectible and cost-effective saliva-derived exosome samples, this approach provides a feasible technical strategy for community-level screening (47).

Furthermore, chemical imaging technology offers an alternative to label-free histopathology. Confer et al. (72) employed quantum cascade laser-based discrete frequency infrared (DFIR) chemical imaging combined with dark-field visible imaging, achieving a diagnostic accuracy of 94.5% compared to the gold standard. This workflow based on chemical imaging holds promise for enhancing the efficiency and accuracy of clinical precancerous diagnosis in oral cancer. Despite its superior performance, such devices are currently expensive and complex to operate, mostly remaining in the proof-of-concept stage, with widespread clinical adoption still requiring time.

### 3.4 Clinical data and prognostic modeling

Clinical data and prognostic modeling primarily utilize large-scale clinical databases (e.g., NCDB, SEER) along with demographic and pathological clinical factors for machine learning modeling. The core objectives are not imaging diagnosis, but rather risk stratification, prognostic assessment, and treatment decision support.

In the prediction of occult metastasis, Bur et al. (27) developed a machine learning algorithm using NCDB data to predict occult lymph node metastasis in early-stage oral cancer (AUC = 0.840), demonstrating superior performance compared to traditional tumor invasion depth (DOI) models, thereby helping to avoid unnecessary neck lymph node dissection. For recurrence and survival prediction, Fatapour et al. (35) constructed a gradient boosting machine model based on the SEER database, achieving an 81.8% accuracy rate in predicting 5-year recurrence in tongue cancer. Kang et al. (61) integrated clinical variables (e.g., lifestyle, TNM staging) into a deep neural network model, with a concordance index of 0.888, outperforming traditional staging methods.

Risk stratification is another key focus in this category. Adeoye et al. (71) utilized a machine learning model to stratify patients with oral leukoplakia and lichenoid mucositis for malignant transformation risk, achieving an accuracy rate of 94%-97%. External validation demonstrated that the predictive factor model exhibited superior sensitivity (0.96) compared to the binary oral epithelial dysplasia risk stratification system (0.82) in identifying patients at risk of malignant transformation. These findings suggest that machine learning-based models may be applicable in various clinical settings for stratifying patients according to malignant transformation risk, thereby addressing the limitations of imaging modalities that focus solely on localized lesions.

### 3.5 General analysis of data input modes

**Figure 2:**
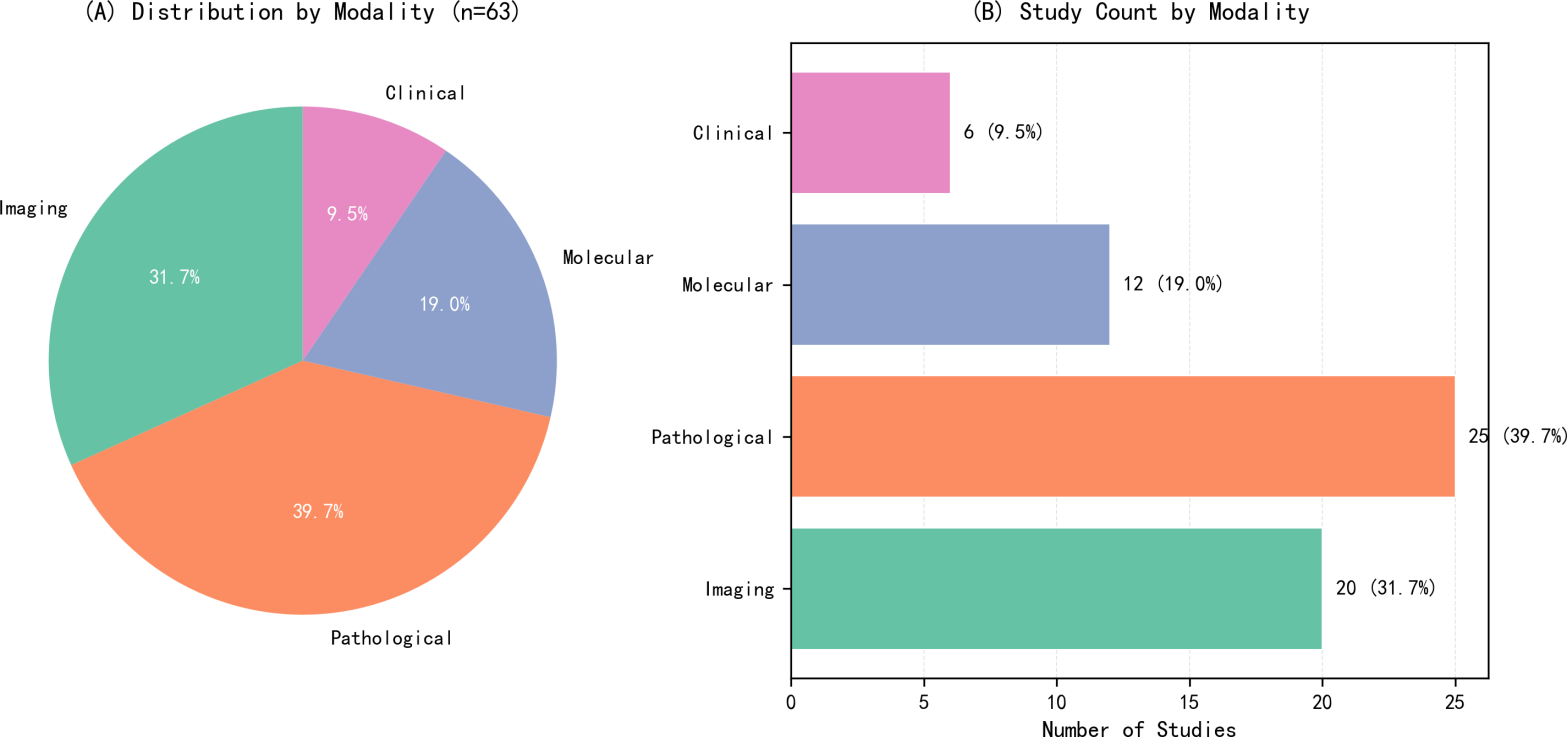
Overview of included studies by data modality. **(A)** Pie chart showing the percentage distribution of 63 studies across four modalities: Imaging (31.7%), Pathological (39.7%), Molecular (19.0%), and Clinical (9.5%). **(B)** Bar chart displaying the absolute number of studies for each modality.

**Figure 3:**
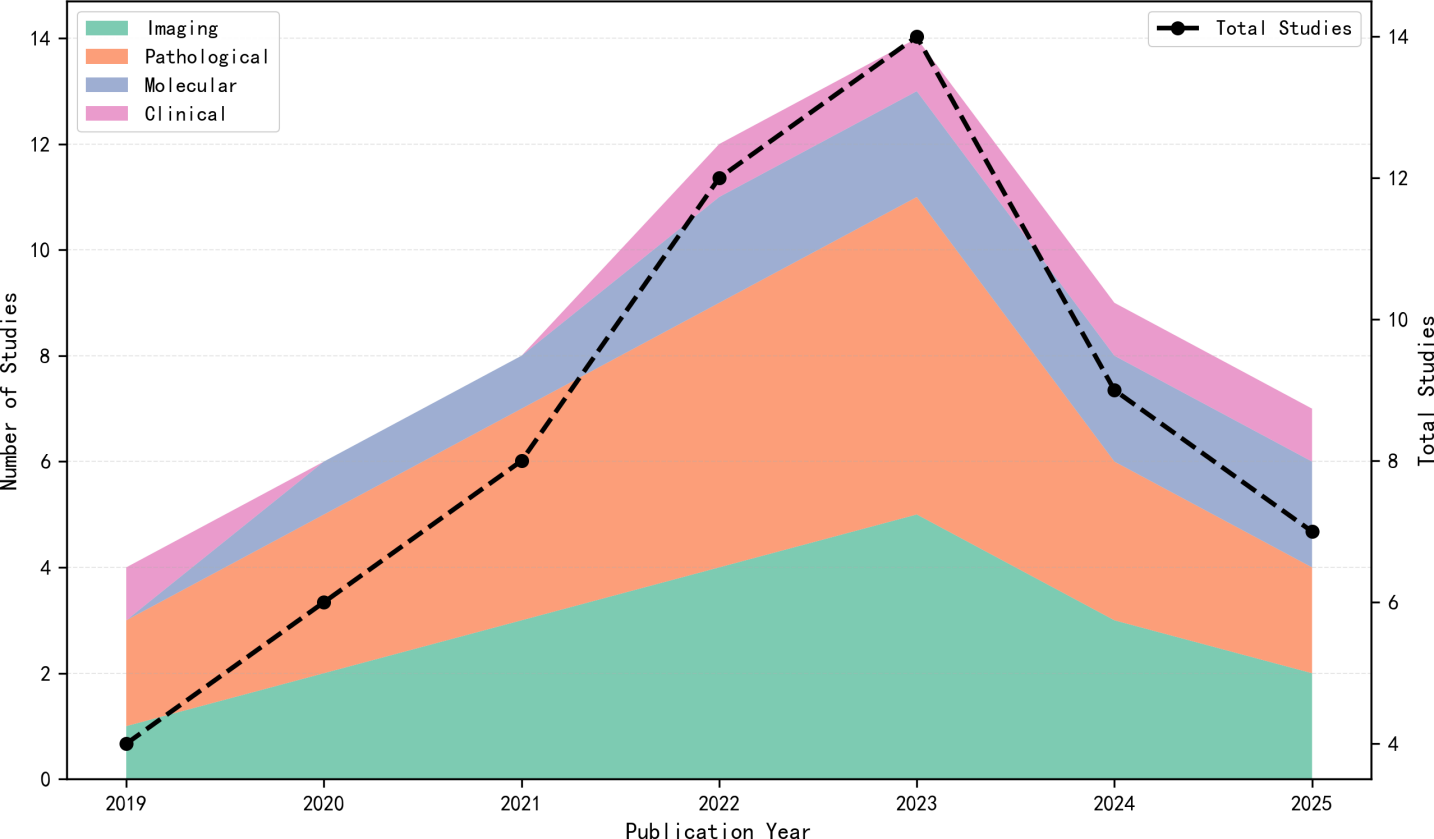
Annual publication trends by modality (2019–2025).

**Figure 4:**
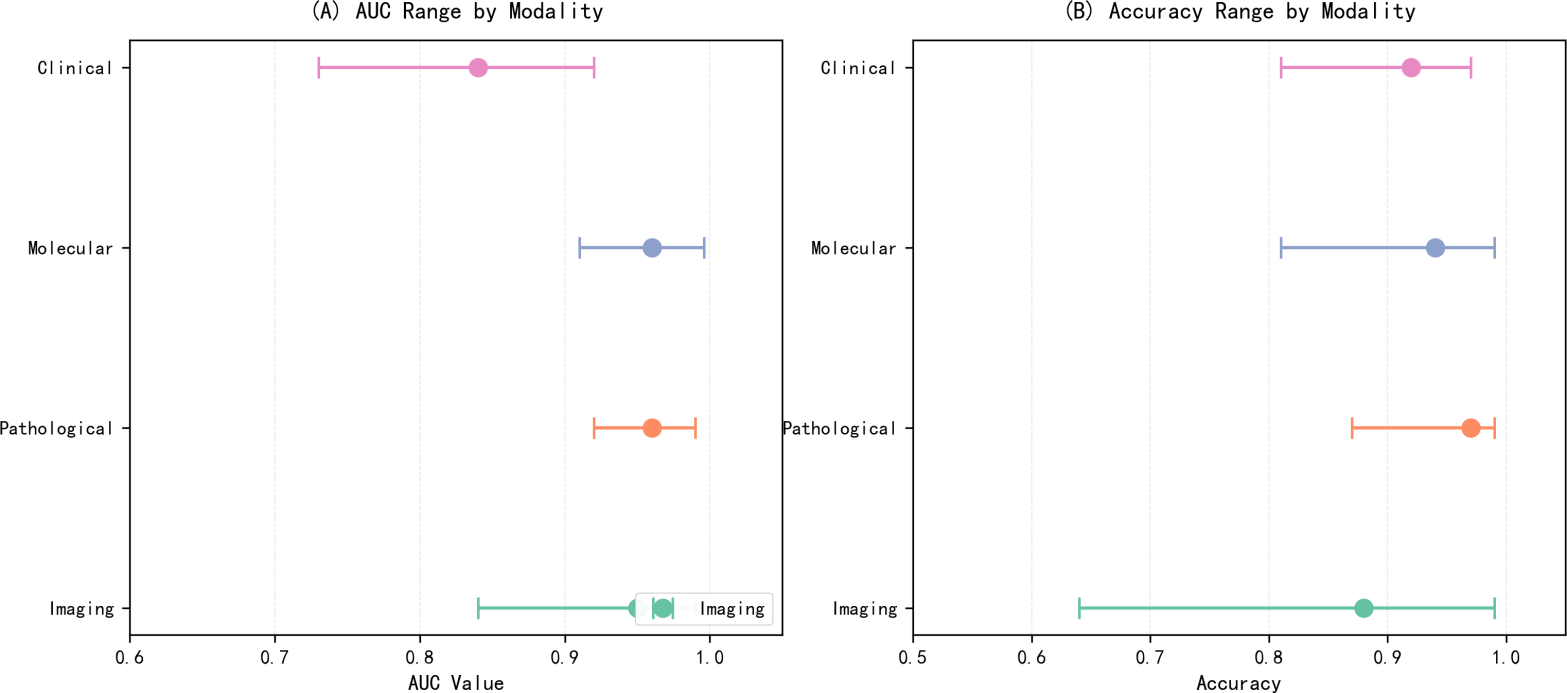
Diagnostic performance of AI models across different data modalities. **(A)** Range of AUC (Area Under the Receiver Operating Characteristic Curve) values for Clinical, Molecular, Pathological, and Imaging modalities. **(B)** Range of accuracy values for each modality. Dots represent mean values, and horizontal lines indicate the range of reported values.

Among the 63 included studies, the data input modalities ranked by prevalence from highest to lowest were: pathological imaging, optical and radiological imaging, molecular and cellular imaging, and clinical data and prognostic modeling, as shown in **Figure 2**. This indicates that AI is currently primarily applied to optical radiological imaging and pathological imaging in early oral cancer screening. However, since 2023, the number of publications in these two fields has decreased annually, while those in molecular and cellular imaging and clinical data and prognostic modeling have increased year by year, as illustrated in **Figure 3**. This suggests that the research focus of artificial intelligence in early oral cancer screening has shifted from macroscopic optical and pathological domains to microscopic molecular and cellular domains or clinical data. Within each data input modality, the AUC values and accuracy rates also varied, as depicted in **Figure 4**. Among the four data input modalities, the pathological imaging domain exhibited the highest average AUC values and accuracy rates across different studies, demonstrating the greatest stability. Combined with the highest number of publications in this field, this confirms that AI technology is most maturely applied in pathological imaging.

## 4 Evolution of algorithm technology

This section systematically reviews the application trajectory of artificial intelligence technology in early oral cancer screening from four dimensions: evolution of algorithmic architecture, optimization of learning strategies, task-adaptive features, and clinical translation adaptability. Complementing the aforementioned data modality analysis, this section focuses on methodological advancements at the algorithmic level and their alignment with clinical needs.

### 4.1 Evolutionary trajectory of core algorithm architecture

The algorithmic development of AI-assisted diagnosis in oral cancer exhibits a clear technical iteration path. Early studies (2019–2021) predominantly employed mature convolutional neural network architectures as the backbone for feature extraction. Warin et al. (20, 21) constructed classification models based on DenseNet-121 and ResNet-50, achieving an AUC of 95% and a sensitivity of 100% in oral photo-based OPMD screening, validating the reliability of classical CNNs in binary classification tasks. Similarly, Ahmad et al. (25) and Fati et al. (58) utilized pre-trained models such as Xception and InceptionResNetV2 for transfer learning, combined with SVM classifiers, to achieve early diagnosis of histopathological images with an accuracy rate of 97%.

After 2022, research focus gradually shifted toward lightweight and edge deployment. Jubair et al. employed a pre-trained EfficientNet-B0 to construct a lightweight CNN, achieving an accuracy of 85.0% on 716 clinical images, providing a feasible solution for screening in resource-constrained environments (3). Desai et al. further compared cloud-based models (DenseNet201, 20M parameters) with local models (FixCaps, 0.83M parameters), demonstrating that the latter significantly reduced computational load while maintaining an accuracy of 83.8%, reflecting active algorithm design adaptation to clinical scenarios (22). Kabir et al. proposed LightSE-MobileViT, integrating a lightweight CNN backbone with a Transformer encoder, achieving an accuracy of 98.39% and an ROC-AUC of 1.00 on clinical validation datasets, marking substantial progress in the co-optimization of lightweight and high-performance architectures (45).

Recent studies (2024–2026) have begun to explore the application potential of Transformers and hybrid architectures in complex tasks. Tafala et al. (29) proposed DeepPatchNet, which integrates DeepLabV3+ with ConvMixer, achieving an F1 score of 86.75% in histopathological image classification, outperforming single ViT or CNN models. Pham (64) effectively mitigated class imbalance by training SVM classifiers with features fused from InceptionResNet-v2 and ViT. Notably, hybrid strategies (CNN feature extraction + traditional machine learning classification) remain practical: Ahmad et al. (25) and Zafar et al. (59) improved OSCC diagnostic AUC to 96.80%-97.78% by integrating deep features with manually extracted features such as GLCM and LBP, combined with PCA dimensionality reduction and SVM classification, demonstrating the advantages of feature engineering and deep learning synergy in data-limited scenarios.

**Figure 5:**
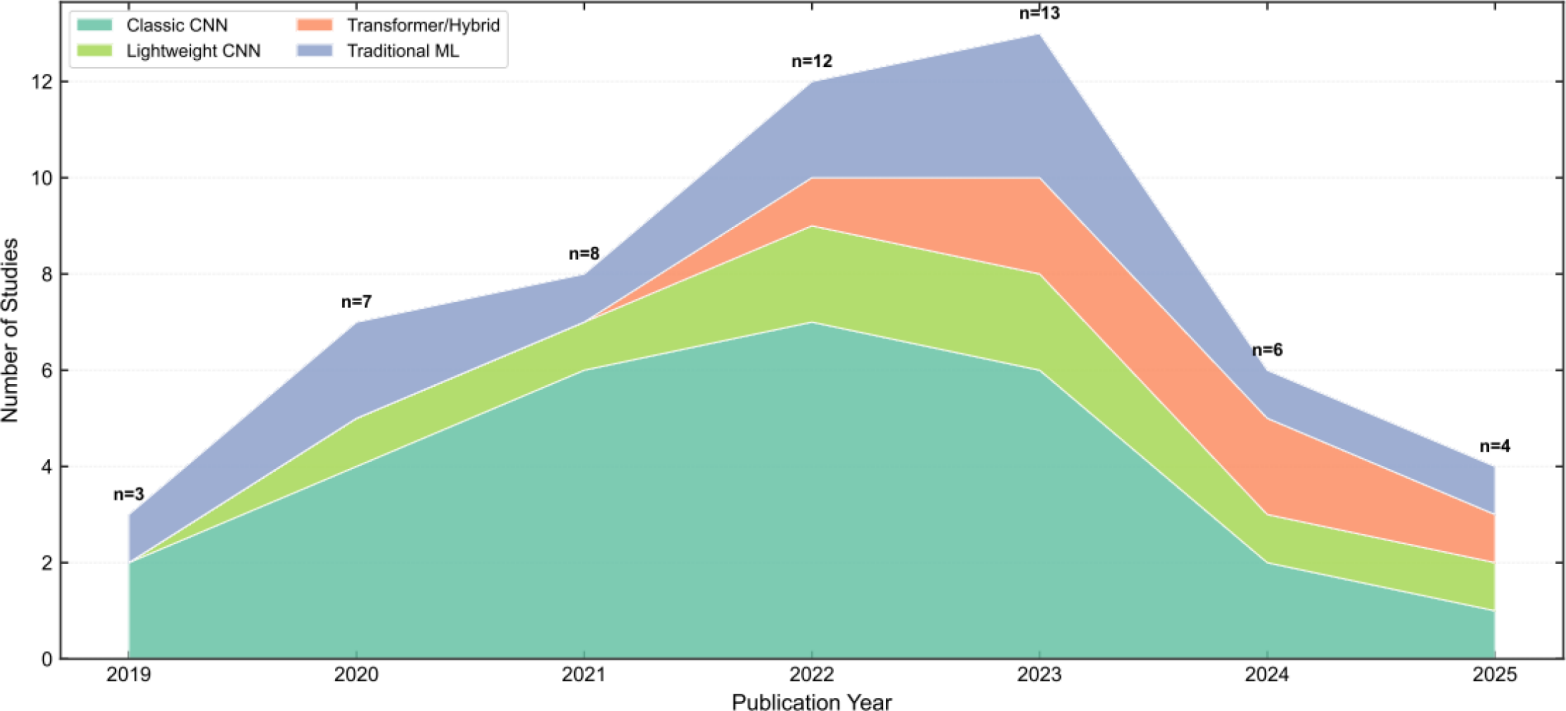
Algorithm architecture evolution trend(2019-2025).

**Figure 5** illustrates the evolutionary trajectory of AI core algorithm architectures. The development of AI algorithms can be categorized into three distinct phases: (1) From 2019 to 2021, classical CNN models dominated the field. (2) During 2021-2023, Transformer architectures and hybrid architectures emerged, while classical CNN models remained prevalent. (3) From 2023 to 2025, the proportion of classical CNN models declined, with all four algorithm types achieving significant advancements.

### 4.2 Study learning strategy optimization and data efficiency improvement

The high cost of medical image annotation and limited sample size have prompted researchers to develop various efficient learning strategies to enhance model generalization capabilities.

Transfer learning remains the mainstream approach. Over 80% of the studies in the literature employed ImageNet pre-trained weights for fine-tuning (3, 21, 44, 45). Monani et al. (63) achieved an ROC-AUC of 0.9082 on the test set by implementing a personalized transfer learning CNN architecture. Afify et al. (66) combined deep transfer learning with Grad-CAM to achieve 100% accuracy on 100× magnified pathological images, validating the effectiveness of pre-training strategies in small-sample scenarios.

To address the scarcity of labeled data, small-sample learning and weakly supervised methods have gained attention. Guo et al. (60) proposed a dual-feature extractor prototype network, which outperforms traditional prototype networks on histopathological datasets by separating the extraction paths of prototype features and query features. Bashir et al. (18) employed a weakly supervised approach to train a malignant transformation prediction model on whole-slide images (WSI), achieving an AUROC of 0.73-0.78 with only image-level labels, significantly reducing reliance on pixel-level annotations.

Class imbalance is a prevalent challenge in oral cancer screening. Most studies employ strategies such as SMOTE over-sampling (63), weighted Fisher score (17), or hybrid feature fusion (25, 58) to mitigate this issue. Kabir et al. (45) enhanced the original 131 images to 981 through data augmentation, effectively improving the model’s generalization capability. Zafar et al. (59) introduced an improved Haris Hawks optimization algorithm for feature selection, maintaining an 899-dimensional feature vector while elevating the OSCC classification rate to 97.78%, demonstrating the value of synergy between feature engineering and optimization algorithms.

### 4.3 Task-oriented model adaptation features

Algorithm selection should be tailored to specific clinical tasks, as different tasks exhibit distinct requirements for precision, speed, and interpretability.

Image classification tasks primarily focus on benign/malignant differentiation and precancerous lesion grading. CNN and lightweight Transformer architectures are widely applied: LightSE-MobileViT (45), DenseNet (22), and ConvNeXt (44) have demonstrated excellent performance in clinical image classification. Goswami et al. (53) employed LightGBM combined with manual features to achieve a 99.25% test accuracy rate in precancerous stage classification of white-light images, indicating that traditional machine learning methods remain competitive when feature engineering is sufficiently robust.

The task of object detection and segmentation focuses on lesion localization and boundary delineation. The YOLO series (v5/v7/v8) is widely used for lesion triage due to its fast inference speed (38, 43, 67), while Yu (67) achieved an F1 score of over 0.84 using the YOLOv7-D6 model for malignant lesion recognition. Faster R-CNN demonstrates stable performance in scenarios requiring higher precision (4, 20, 21). In segmentation tasks, U-Net and its variants dominate: Pushparathi et al. (17) improved the U-Net classifier to achieve an accuracy rate of 99.54%, and Musulin et al. (47) implemented epithelial-mesenchymal tissue segmentation using DeepLabv3+ combined with Xception_65, achieving a mean inter-observer uncertainty (mIOU) of 0.878.

Prognosis and risk prediction tasks often employ ensemble learning or deep neural networks to process structured clinical data. Bur et al. (27) developed a decision forest algorithm based on NCDB data, achieving an AUC of 0.840 for predicting occult lymph node metastasis, which outperformed traditional DOI models. Adeoye et al. (71) integrated 26 clinical variables to construct an ML risk model, demonstrating an accuracy rate of 97% in predicting malignant transformation of oral leukoplakia and an external validation sensitivity of 0.96, highlighting the value of multi-factor integration in risk stratification.

### 4.4 No clinical suitability and reliability assessment

There exists a gap between laboratory metrics of algorithm performance and clinical translation requirements, with generalization ability, interpretability, and operational efficiency becoming key determinants of practical applicability.

Interpretability is a core component in establishing clinical trust. Grad-CAM has become a mainstream visualization tool (29, 44, 66) that highlights diagnostic regions through heat maps. Parola et al. (76) further proposed an information deep learning (IDL) combined with case reasoning paradigm, achieving an 85% accuracy rate on “imperfect images” containing labeled noise and artifacts (superior to the 77% of pure DL), with generated interpretations more aligned with clinical cognitive logic, providing new insights for the clinical adaptation of XAI.

Operational efficiency directly impacts the feasibility of real-time screening. Guedes et al. (19) and Lepper et al. (32) validated the efficacy of AI systems in cytological analysis, demonstrating a reduction in analysis time by 16.6 to 100-fold compared to manual methods, thereby ensuring efficiency for large-scale screening.

Generalization capability and external validation remain weak links in current research. Most literature is based on single-center retrospective data, with insufficient external validation. Talwar et al. (37) found that when test set images were captured by untrained personnel, the model’s F1 score dropped from 0.84 to 0.73, indicating that image quality variation poses challenges to model robustness. Adeoye et al. (42, 71) conducted multicenter or geographic validation, where Adeoye et al. (42)’ s DL model achieved a balanced accuracy rate of 76.4% in external validation, surpassing the 92.3% performance of human raters, providing positive evidence for cross-center generalization. Yang et al. (69) further pointed out that tobacco use significantly interferes with screening algorithm performance (sensitivity for smokers: 80.0% vs. non-smokers: 62.1%), suggesting that clinical risk factors should be incorporated into algorithm design considerations.

### 4.5 Summary of algorithmic technical features

To systematically present the legal technical characteristics of the included literature, **Table 2** summarizes the main algorithm categories, representative models, application scenarios, core advantages, and limitations.

**Table 2:** Technical characteristics and performance analysis of AI-assisted Screening algorithms for oral cancer.

Algorithm category	Representation model/architecture	Main application scenarios	Core advantage	Key limitations	Representative document
classics CNN	DenseNet-121/169, ResNet-50/101, EfficientNet-B0/B3	Classification of clinical photographs, preliminary screening of pathological images	The model is mature with rich pre-training weights, and its binary classification AUC is often>0.95.	Limited sensitivity, potential performance degradation in complex multi-classification tasks, and weak interpretability.	(3, 4, 20, 21, 44, 49, 50, 56, 58, 63, 77)
lightweight network	MobileNetV2/V3, FixCaps, LightSE-MobileViT	Mobile screening, edge computing deployment	Fewer parameters (as low as 0.83M) and faster inference (<300ms per image))	Model capacity is limited, and the generalization ability of complex background needs improvement.	(3, 22, 44, 45, 66)
object detection/segmentation	Faster R-CNN, YOLOv4/v5/v7, U-Net, DeepLabV3	Lesion localization, border delineation, tissue segmentation	Export spatial location information and support pixel-level analysis	Precise labeling is required, as the detection accuracy of small lesions is limited.	(4, 20, 21, 38, 40, 44, 47, 52, 67)
Transformer/Mixed Architecture	ViT, Swin, ConvMixer, DeepPatchNet	Pathological multi-classification and multimodal fusion	Capture global context for complex tasks	High data demand and high computing resource consumption	(29, 37, 45, 64, 66)
Traditional ML/hybrid methods	SVM, Random Forest, LightGBM	Small-sample learning, clinical data modeling	Small data is robust and highly interpretable	High-dimensional images exhibit weak extraction capability due to reliance on feature engineering.	(25, 27, 34, 39, 51, 56, 57, 63, 68, 71)
special learning strategy	TL, Small sample learning, Weak supervision, Data augmentation	Data scarcity scenarios and category imbalance	Addressing the challenges of difficult labeling and limited samples to enhance generalization capabilities	Unstable training and complex parameter tuning	(11, 18, 25, 37, 45, 60, 63, 66)
comprehensibility AI	Grad-CAM, Case-Based Reasoning, IDL	Enhance transparency and support clinical decision-making	Visualize decision-making basis and establish clinical trust	Reliability explanation requires verification and may increase computational overhead	(11, 29, 44, 66, 76)

## 5 Trend analysis and future outlook

### 5.1 Trends in algorithm architecture development

The algorithmic architecture of early-stage (2019–2021) AI diagnostic models for oral cancer primarily relied on classical convolutional neural networks (CNNs), such as DenseNet, ResNet, and the VGG series, which demonstrated high stability in binary classification tasks, with some models achieving AUC values exceeding 0.95 (20, 21). However, with increasing demands for real-time performance and deployment environments in clinical scenarios, recent research (2022–2026) has progressively shifted toward lightweight and high-performance architectures. On one hand, lightweight architectures like MobileNet and EfficientNet have been widely adopted for resource-constrained primary screening scenarios, with some models maintaining an accuracy rate of 83.8% even with reduced parameter counts to 0.83 million, achieving a transition from cloud to edge computing (3, 22). On the other hand, to capture global contextual information in pathological images, Transformer and hybrid architectures (e.g., MobileViT, ConvMixer) have been applied to complex classification tasks. The LightSE-MobileViT model achieved an accuracy rate of 98.39% on clinical validation datasets, demonstrating superior feature extraction capabilities compared to traditional CNNs (45). Furthermore, to address the challenges of high annotation costs and class imbalance in medical data, small-sample learning, weakly supervised methods, and hybrid feature fusion strategies (e.g., CNN combined with SVM or GLCM texture features) have become crucial approaches to enhance model generalization performance. Some hybrid models have achieved an AUC improvement of 99.52% in histopathological diagnosis (11, 25, 60).

### 5.2 Trends in multimodal fusion and detection technology

**Figure 6:**
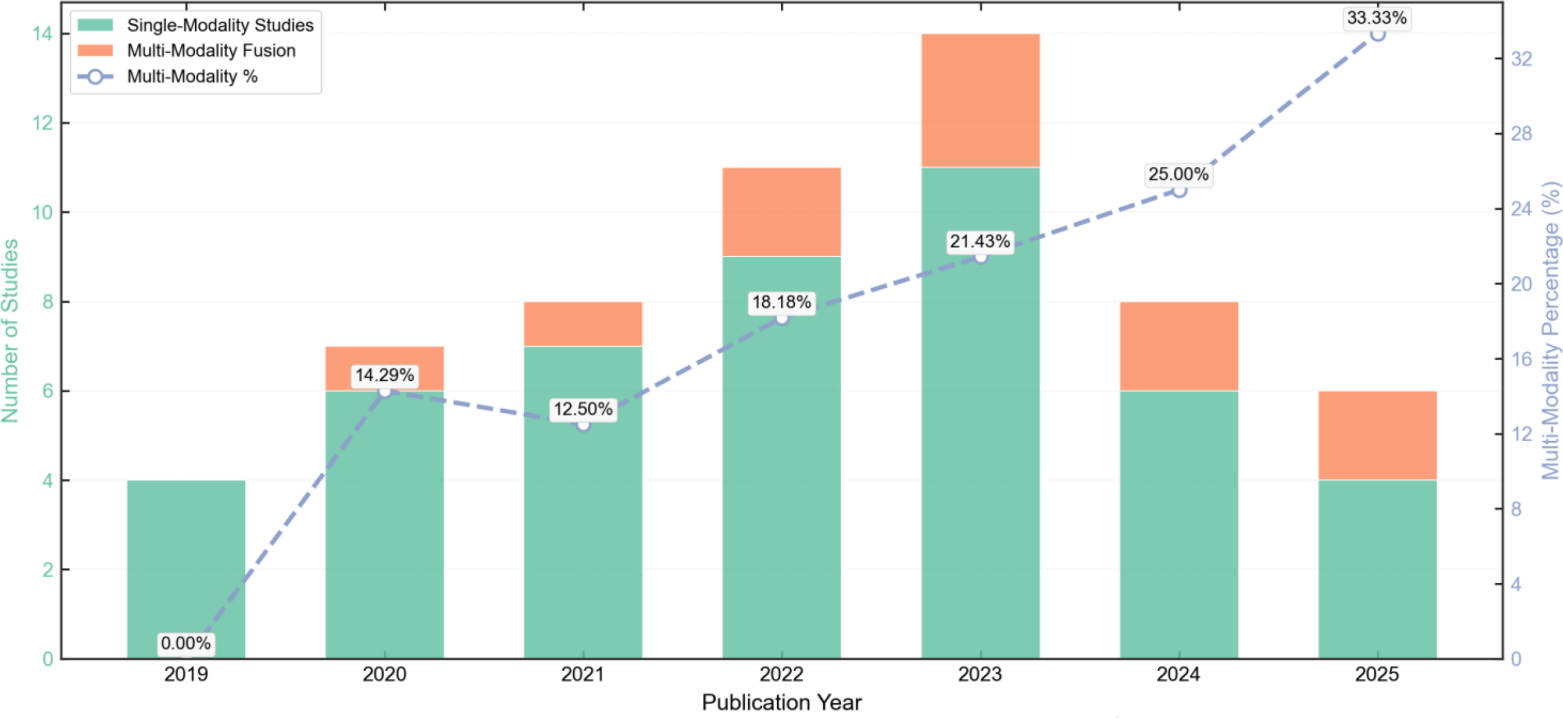
Evolution of multi-modality fusion research(2019-2025).

In terms of data modalities, single-modality optical imaging remains dominant, but multimodal fusion has become a key trend for enhancing diagnostic robustness. Current research primarily focuses on four categories: visible light photographs, optical coherence tomography (OCT), spectral imaging, and histopathological images. Visible light photographs, due to their low cost and high accessibility, are widely used for initial screening, but they are susceptible to interference from environmental factors such as lighting and angle, often leading to a decline in external validation performance (37, 61). In contrast, OCT and spectral imaging (e.g., Raman spectroscopy, impedance spectroscopy) can provide information on tissue microstructure and molecular fingerprints, with non-invasive detection accuracy reaching 91%-99%, demonstrating potential to replace some invasive biopsies (23, 24, 31, 55). Notably, multimodal deep learning processes that integrate patient metadata (e.g., age, smoking history) with image data are emerging. Such models mimic the comprehensive diagnostic logic of clinicians and outperform single-image data in early detection of potential malignancies (66). Meanwhile, the development of smartphone-based dual-mode devices (combining fluorescence imaging and spectroscopy) and point-of-care testing (POCT) tools has further narrowed the gap between laboratory technologies and clinical practice (73). As shown in **Figure 6**, since 2021, the proportion of articles published annually using multimodal fusion technology has been increasing year by year.

### 5.3 Clinical translation challenges and future directions

Despite the outstanding performance of technical indicators, the clinical translation of AI-based oral cancer screening technology still faces significant challenges. Firstly, the evidence level is generally low, with over 90% of studies being single-center retrospective designs, lacking external validation and prospective multicenter clinical trials, which raises doubts about the generalizability of the models in real-world settings (43, 63). Secondly, the lack of algorithmic interpretability constrains the establishment of clinical trust. Although explainable AI (XAI) techniques such as Grad-CAM and case reasoning have been introduced, whether the generated explanations align with clinical cognitive logic still requires further validation (76). Additionally, confounding factors such as tobacco use significantly impact the performance of screening algorithms, suggesting that future model architectures should incorporate more clinical variables to enhance robustness (69).

Future research should focus on the following directions: First, conduct large-scale, multicenter prospective validation to establish standardized data collection and annotation protocols, and evaluate the generalizability of models across different healthcare systems. Second, deepen interpretability studies by developing “white-box” models that align with clinical decision-making logic, thereby enhancing physicians’ trust in AI-assisted diagnosis. Third, promote multimodal data integration by consolidating imaging, pathological, genomic, and clinical follow-up data to build a comprehensive risk prediction and prognostic evaluation system. Fourth, strengthen clinical workflow integration by developing AI tools compatible with existing hospital information systems to achieve seamless transition from screening to diagnosis and treatment planning. Only through dual drivers of technological optimization and clinical validation can artificial intelligence truly realize early diagnosis and treatment of oral cancer, thereby reducing the global disease burden.

## 6 Conclusion

Based on the 63 original studies included, the field of AI-assisted screening for oral cancer demonstrates three major evolutionary trends: At the algorithmic level, model architectures have progressed from traditional CNNs to hybrid frameworks integrating lightweight networks and Transformers, with transfer learning and few-shot learning strategies effectively alleviating constraints posed by limited medical imaging data (20, 45, 60). At the modal level, while monomodal optical image-based screening remains predominant, multimodal fusion approaches—such as combining imaging with clinical metadata or integrating spectroscopy with imaging—are emerging as pivotal pathways for enhancing diagnostic robustness (66, 73). At the application level, the research focus is gradually shifting from technical performance validation toward integration into clinical workflows, with real-time detection and risk stratification representing promising new directions (19, 71).

Current transformational challenges are primarily focused on three aspects: First, the quality of evidence remains constrained, as over 90% of studies are based on single-center retrospective designs, and insufficient external validation raises concerns regarding model generalizability (37, 43). Second, deficient model interpretability limits clinical adoption; although visualization techniques such as Grad-CAM have been employed, their explanatory rationale requires further validation to ensure alignment with clinical reasoning (76). Third, confounding factors—such as tobacco use and variability in image quality—that influence algorithmic performance have not been systematically incorporated into model architectures (69).

Overall, AI represents not merely a replacement for conventional screening methodologies, but a novel developmental paradigm for early oral cancer detection. Future progress will depend on establishing a closed-loop framework encompassing multi-center data sharing, methodological innovation, and clinical pilot implementation. Simultaneously, enhanced collaboration with public health and policy-making institutions will be essential to transition AI from a purely technological pursuit to a tool effectively serving disease prevention and control systems. As this evolution proceeds, AI is anticipated to evolve from an auxiliary diagnostic technology into a foundational component supporting the precision prevention and management of oral cancer.
